# CME ACTIVITY: Preventing and Controlling Emerging and Reemerging Transmissible Diseases in the Homeless

**DOI:** 10.3201/eid1409.082041

**Published:** 2008-09

Medscape, LLC is pleased to provide online continuing medical education (CME) for this journal article, allowing clinicians the opportunity to earn CME credit. Medscape, LLC is accredited by the Accreditation Council for Continuing Medical Education (ACCME) to provide CME for physicians. Medscape, LLC designates this educational activity for a maximum of 0.75 *AMA PRA Category 1 Credits*™. Physicians should only claim credit commensurate with the extent of their participation in the activity. All other clinicians completing this activity will be issued a certificate of participation. To participate in this journal CME activity: (1) review the learning objectives and author disclosures; (2) study the education content; (3) take the post-test and/or complete the evaluation at **http://www.medscape.com/cme/eid**; (4) view/print certificate.

## Learning Objectives

Upon completion of this activity, participants will be able to:

- Describe practices to reduce the burden of HIV and hepatitis infection among the homeless.
- Identify how to screen for tuberculosis and treat tuberculosis in homeless settings.
- Describe the problem of scabies and body louse infections among the homeless.
- Specify the burden of illness associated with *Bartonella quintana* among the homeless and how to treat this infection.

## Editor

**Anne Mather**, Technical Writer-Editor, *Emerging Infectious Diseases*. *Disclosure: Anne Mather has disclosed no relevant financial relationships.*

## CME AUTHOR

**Charles P. Vega, MD**, Associate Professor; Residency Director, Department of Family Medicine, University of California, Irvine. *Disclosure: Charles P. Vega, MD, has disclosed that he has served as an advisor or consultant to Novartis, Inc.*

## AUTHORS

Disclosures: **Sékéné Badiaga, MD**; **Didier Raoult, MD, PhD**; and **Philippe Brouqui, MD, PhD**, have disclosed no relevant financial relationships.

## Earning CME Credit

To obtain credit, you should first read the journal article. After reading the article, you should be able to answer the following, related, multiple-choice questions. To complete the questions and earn continuing medical education (CME) credit, please go to **http://www.medscape.com/cme/eid**. Credit cannot be obtained for tests completed on paper, although you may use the worksheet below to keep a record of your answers. You must be a registered user on Medscape.com. If you are not registered on Medscape.com, please click on the New Users: Free Registration link on the left hand side of the website to register. Only one answer is correct for each question. Once you successfully answer all post-test questions you will be able to view and/or print your certificate. For questions regarding the content of this activity, contact the accredited provider, CME@medscape.net. For technical assistance, contact CME@webmd.net. American Medical Association’s Physician’s Recognition Award (AMA PRA) credits are accepted in the US as evidence of participation in CME activities. For further information on this award, please refer to http://www.ama-assn.org/ama/pub/category/2922.html. The AMA has determined that physicians not licensed in the US who participate in this CME activity are eligible for *AMA PRA Category 1 Credits*™. Through agreements that the AMA has made with agencies in some countries, AMA PRA credit is acceptable as evidence of participation in CME activities. If you are not licensed in the US and want to obtain an AMA PRA CME credit, please complete the questions online, print the certificate and present it to your national medical association.

### Article Title: Preventing and Controlling Emerging and Reemerging Transmissible Diseases in the Homeless

## CME Questions

1. Which of the following statements about reducing the risk for incident HIV and hepatitis infection among homeless individuals is most accurate?
  A. Older homeless adults should be targeted for HIV prevention
  B. Education and skills training can reduce the practice of unprotected sex among homeless women
  C. Homeless people are less likely than other intravenous drug users to complete hepatitis B virus (HBV) vaccination
  D. Homeless people should never receive the accelerated HBV vaccination schedule
2. Which of the following statements about tuberculosis and airborne diseases among the homeless is most accurate?
  A. Most tuberculosis infections among homeless individuals are reactivations of established disease
  B. Sputum testing detects >90% of patients with tuberculosis
  C. Screening for tuberculosis with chest x-ray may be the most cost-effective approach
  D. Directly observed therapy in the acute hospital setting is associated with the highest completion rates
3. Which of the following statements about scabies and body louse infections in the homeless is most accurate?
  A. The body louse is an efficient vector for multiple species of bacteria
  B. Ivermectin is ineffective in treating scabies
  C. A treatment regimen of clothing change and medical treatment has been demonstrated to eliminate scabies from a homeless shelter
  D. The prevalence of body lice among sheltered homeless is approximately 5%
4. Which of the following statements about* Bartonella quintana *infection is most accurate?
  A. *B quintana* is the most common louse-borne disease reported among urban homeless
  B. *B quintana* does not cause endocarditis
  C. Body lice are the natural reservoir for *B quintana*
  D. Cefixime should be used for serious infections with *B quintana*

### Activity Evaluation

**Table:** 

**1. The activity supported the learning objectives.**				
Strongly Disagree				Strongly Agree
1	2	3	4	5
**2. The material was organized clearly for learning to occur.**				
Strongly Disagree				Strongly Agree
1	2	3	4	5
**3. The content learned from this activity will impact my practice.**				
Strongly Disagree				Strongly Agree
1	2	3	4	5
**4. The activity was presented objectively and free of commercial bias.**				
Strongly Disagree				Strongly Agree
1	2	3	4	5

